# Effects of Dog Assisted Therapy for Adults with Autism Spectrum Disorder: An Exploratory Randomized Controlled Trial

**DOI:** 10.1007/s10803-019-03971-9

**Published:** 2019-03-21

**Authors:** Carolien Wijker, Ruslan Leontjevas, Annelies Spek, Marie-Jose Enders-Slegers

**Affiliations:** 1 GGZ Oost Brabant, P.O. Box 3, 5427 ZG Boekel, The Netherlands; 2 Faculty of Psychology and Educational Sciences, Open University of the Netherlands, Heerlen, The Netherlands; 3 Department of Primary and Community Care, Center for Family Medicine, Geriatric Care and Public Health, Medical Centre, Radboud University Nijmegen, Nijmegen, The Netherlands; 4 Autism Center of Expertise, Eemnes, The Netherlands

**Keywords:** Autism, Adults, Animal assisted therapy, Dogs, Stress-related problems

## Abstract

Effective treatments of highly prevalent stress-related outcomes such as depression and anxiety are understudied in adults with autism spectrum disorder (ASD). A randomized controlled trial with baseline, post-intervention, and 10-week follow-up, that explores the effects of animal assisted therapy (AAT) was conducted. In total, 53 adults with ASD with normal to high intelligence were randomized in an intervention (N = 27) versus waiting list control group (N = 26). The remarkable adherence to the therapy program by study participants and the program’s clinically relevant effects indicate that AAT with dogs can be used to reduce perceived stress and symptoms of agoraphobia, and to improve social awareness and communication in adults with ASD with normal to high intelligence.

Stress-related mental health problems such as depression and anxiety are very common in adults with autism spectrum disorder (ASD), affecting up to 77% of this population (Joshi et al. 2013). Stress is strongly associated with depression and anxiety (Vreeburg et al. 2010), premature mortality and poor health outcomes (Slavich 2016), and severity of ASD traits, which include problems in social interaction and communication (Hirvikoski and Blomqvist 2015). To date, research on effective interventions that aim to reduce stress and stress-related outcomes in people with ASD or to improve their social interaction and communication has been very limited (Damiano et al. 2014). A few randomized controlled trials (RCTs) conducted on interventions in ASD suggest that cognitive behavioral therapy (CBT) and mindfulness-based stress reduction (MBSR) can be effective for reducing depression and anxiety (Sizoo and Kuiper 2017). This effect may partially be explained by physical stress reduction, e.g. measured using markers such as salivary cortisol (Vreeburg et al. 2010). To the best of our knowledge, no RCTs have been reported that directly target perceived stress in the adult ASD population.

Several studies not specific to adults with ASD have shown that physical interactions with animals reduce stress levels (Beetz et al. 2012). In children with ASD, animal assisted therapies (AAT) (i.e., interventions that incorporate trained animals) have also shown promising results for stress-related outcomes (O’Haire 2013). Improvements in social interaction and communication have also been reported (Berry et al. 2013; Gabriels et al. 2015). AAT may be especially well suited to people with ASD because animals communicate non-verbally, which may be a less stressful form of interaction than a conversation with a therapist involving metacognitive and introspective aspects (Verheggen et al. 2017). It has been hypothesized that in therapeutic settings, animals act as social catalysts, causing patients to become more willing to communicate with their social environment which in turn facilitates improvements in social interaction and communication (Gabriels et al. 2015). Although the described effects of AAT (mostly with dogs) in children with ASD are promising, it is not clear whether these results can be generalized to adults. Importantly, AAT studies in children report a number of limitations such as small sample sizes, limited or no verification of the ASD diagnosis, limited descriptions of the intervention, and lack of control groups, randomization, and validated outcome measures (O’Haire 2013). To the best of our knowledge, no interventions that include human–animal interactions have ever been reported in adults with ASD.

To summarize evidence on animal assisted therapy (AAT) in adults with autism spectrum disorder (ASD), we conducted a systematic search of PubMed looking specifically for reports published in Dutch and English between January 1, 2000 and December 31, 2015. Studies were eligible if they were treatment effect studies, included people with ASD, and contained at least one treatment group that incorporated a live animal. We identified a total of 18 studies. The animals in these studies included dogs, horses, guinea pigs, lamas, and dolphins. None of the studies included participants of 18 years or older. We extended the systematic search to reports published between January 1, 1995, and April 16, 2018, and still found no research that included participants with ASD who were 18 years or older.

Our study contributes to the scientific literature by specifically including adults with ASD, by using an appropriate sample size for an explorative effect study, and including a control group, randomization, and valid outcome measures.

Previous studies in children with ASD have shown that AAT offers promising results in terms of stress reduction and improvements in social communication skills. Our study shows that the highly understudied population of adults with ASD can also benefit from AAT in similar ways, including reduction of perceived stress, agoraphobia and improvements in one of the core aspects in ASD, social responsiveness (as reported by proxies).

We considered the effects of AAT in children with ASD (O’Haire 2013; Gabriels et al. 2015) and hypothesized that in adults with ASD, AAT may result in stress reduction, improvements in social responsiveness (social awareness, communication, and motivation), and reduced depressive and anxiety symptoms, which are strongly related to stress (Vreeburg et al. 2010). Considering that stress-reducing effects of dogs were reported in the general population (Beetz et al. 2012) and that dogs are the animals most commonly employed in AAT with children with ASD (O’Haire 2013), our aim was to explore the effects of AAT with dogs in adults with ASD with normal to high intelligence. We focused on self-perceived stress, social responsiveness, and psychological symptoms (such as depression and anxiety symptoms). Furthermore, we looked at AAT’s effects on self-esteem in this same group—an important addition, given that adults with ASD were found to have lower self-esteem than adults without ASD and that self-esteem is strongly negatively correlated with the stress-related outcomes depression and anxiety (Cooper et al. 2017).

## Methods

### Study Design

The randomized controlled trial (RCT) with two arms (the intervention condition and the waiting list control condition) was conducted between January 2015 and July 2017 and had an exploratory character due to the lack of evidence on AAT in adults with ASD. Subjects entered the study at seven pre-planned starting times, with recruitment continuing until 17 February 2017. For each participant, baseline assessment was followed by the post-intervention assessment (T1, 10 weeks post-baseline) and the follow-up assessment (T2, 20 weeks post-baseline). All waiting list controls were offered the option of individual AAT after T2.

Detailed information about recruitment and screening procedures is reported elsewhere (Wijker et al. 2017).

### Participants

All study participants were recruited sequentially in batches from the mental health care organization GGZ Oost Brabant, The Netherlands, which has a psychiatric outpatient center for adults with ASD with normal to high intelligence. Information flyers in the waiting room and verbal information from therapists were used for recruitment. Inclusion criteria were the following: diagnosed with ASD, between 18 and 60 years of age, and an IQ of 80 or above. Because the intervention was developed to reduce perceived stress and comorbid symptoms in ASD, only participants with a score considered to be high on the Perceived Stress Scale (PSS; Cohen and Williamson 1988, scores > 19) and on the Symptom Checklist-90-Revised (SCL-90-R; Derogatis 1994, scores > 132) were included. Exclusion criteria were current psychosis or suicide risk as indicated by the person’s psychologist or psychiatrist, institutionalization, allergy to dogs, fear of dogs, aversion to dogs, and participation in a treatment other than AAT during the study period (psychopharmacological treatment was allowed as long as the medication remained stable during the study). When an ASD diagnosis and/or IQ score was missing, a standardized diagnostic procedure was conducted by a multidisciplinary team, by a combination of the autistic disorder interview-revised (ADI-R) (Lord et al. 1994), a semi-structured interview of the DSM-5 criteria (American Psychiatric Association 2013) and clinical observations. Subjects were enrolled if they fulfilled diagnostic criteria of ASD and scored 80 or above on the total scale of the Dutch version of the Wechsler Adult Intelligence Scale III/IV (Wechsler 1997, 2008).

Participants provided verbal and written informed consent. Participants were made aware both verbally and in writing that they could end their participation and withdraw at any point during the study. The medical ethics committee CMO region Arnhem-Nijmegen, The Netherlands approved the study.

### Randomization and Masking

Using computer-generated random numbers, participants within a batch were randomized blindly by one of the researchers (RL) to one of the arms (the intervention condition or the waiting list control condition) after the baseline measurements. The researcher (RL) who conducted the effect analyses was masked to the condition to which a specific participant was assigned.

### Outcomes

*Perceived stress* was measured using the Perceived Stress Scale (PSS; Cohen and Williamson 1988), which contains ten items rated on a five-point Likert scale ranging from 0 ‘never’ to 4 ‘very often’. A higher total score corresponds with a higher rating in self-perceived stress. The instrument has a good internal consistency and an adequate convergent validity (Cohen and Williamson 1988).

*Psychological and physical symptoms* were measured with the symptom checklist-90-revised (SCL-90-R; Derogatis 1994). The Dutch version of the SCL-90-R contains 90 items divided into eight subscales that measure psychological and physical distress: anxiety, agoraphobia, depression, somatization, cognitive-performance deficits, interpersonal sensitivity and mistrust, hostility, and sleep difficulties. Each item is rated on a five-point Likert scale ranging from 1 ‘none’ to 5 ‘very severe’. A higher total score corresponds with higher levels of self-reported psychological and physical symptoms. The original SCL-90-R and the Dutch version of this instrument have excellent reliability and construct validity (Arrindel and Ettema 2002).

Impairments in *social responsiveness* were measured with the Social Responsiveness Scale for Adults (SRS-A; Noens et al. 2012). The Dutch version of the SRS-A contains 64 items and four subscales: social awareness, social communication, social motivation, and restricted interests and repetitive behavior. Each item is rated on a four-point Likert scale ranging from 1 ‘not true’ to 4 ‘almost always true’. A higher total score corresponds with more impairment in social responsiveness. This questionnaire has two versions: a self-report and an informant-report. The Dutch version has a good internal consistency and test–retest reliability and a sufficient intraclass correlation coefficient for the self-report and informant report (Noens et al. 2012).

*Self-esteem* was measured using the Rosenberg Self-Esteem Scale (RSES; Rosenberg 1965), which contains ten items, each rated on a four-point Likert scale ranging from 1 ‘very untrue’ to 4 ‘very true’. A higher total score corresponds with higher rating of self-reported self-esteem. This instrument has a high validity and test–retest reliability (Franck et al. 2008).

### Procedures

Animal assisted therapy (AAT) is a goal-oriented, (semi-) structured intervention provided by a certified professional incorporating a trained and certified animal (IAHAIO 2014). The therapy goals for children with ASD who participated in previous research on AAT were improving social interaction, verbal and non-verbal communication skills, and reducing physiological stress (O’Haire 2013). The AAT program for this trial was developed by therapists and dog behavioral specialists from the Dutch service dog foundation Stichting Hulphond Nederland and psychologists from the mental health care organization GGZ Oost Brabant who have a specialization in autism. The program had a structured protocol and consisted of 10 weekly one-on-one sessions of 60 min per session. A therapy dog was involved during all the therapy sessions. The therapists providing AAT had a college or university degree in mental health care and were specialized in working with adults with ASD. Additionally, the AAT therapists had completed advanced courses in dog behavior and welfare. The Dutch service dog foundation Stichting Hulphond Nederland provided the therapy dogs. In total, thirteen therapy dogs were involved in the study (two Labradors, four Labrador crossbreeds, one golden retriever, three golden retriever crossbreeds, two poodles, and one German Wirehaired Pointer). All of the participating dogs were trained and tested to work with people, and their mental and physical health care was strictly monitored by the service dog foundation. Detailed information about the AAT program has been described elsewhere (Wijker et al. 2017).

At baseline, immediately post-intervention, and at 10-week follow-up (T0, T1, and T2), participants were asked to fill in questionnaires in the following order: Perceived Stress Scale (PSS), symptom checklist 90-revised (SCL-90-R), Rosenberg Self Esteem Scale (RSES) and Social Responsiveness Scale-Adults (SRS-A). A stimulus-poor laboratory setting was used at the mental health care organization. A research assistant checked for missing items and asked participants to complete the missing items before leaving the assessment room. Participants received the SRS-A informant version and were asked to have a spouse, close family member, or friend complete the questionnaire. The same informants were to be used at all three assessments (T0, T1, and T2). Informants were asked to return the questionnaire during the week following each assessment. When the proxy-report was not returned, it was reported as missing and not included in analyses.

### Statistical Analyses

IBM SPSS Statistics for Windows, Version 21.0 was used for descriptive statistics and building mixed models. Missing items were extrapolated by the mean score of other scale items. In participants with missing items (n = 6), self-report scales (PSS, SCL-90-R, RSES and SRS-A self-reports) had no more than 10% of their items missing. Fourteen SRS-A questionnaires were not returned and therefore were not included in the analyses.

All participants in the intervention group participated in at least nine of the ten therapy sessions and for this reason we did not account for adherence (the number of sessions a participant received) as we had originally planned (Wijker et al. 2017).

To estimate the intervention effect, we used linear mixed models with random intercepts and accounting for repeated measurements within participants.

In line with the exploratory character of the study, both the total scales’ scores (PSS, SCL-90, RSES, and SRS-A) and the scores of the subscales (SCL-90 and the SRS-A) were used as outcomes. Standardized effect sizes (d) were calculated by dividing estimated effects by the standard deviation at baseline; d of < 0.20, between 0.20 and 0.49, between 0.50 and 0.79, and > 0.80 was interpreted respectively as negligible, small, medium, and large (Cohen 1988). In addition, we did not correct for multiple testing and built two models per outcome: (1) with main fixed effects (the intervention condition [yes/no] and the assessment time points [T1 and T2 compared to T0] and (2) a mixed model with the same fixed terms and preplanned covariates age (years), gender (male versus female), having a dog at home at T0 (yes/no), and WAIS total IQ. During the trial, psychotropic medication was changed for some participants, and therefore, additional analysis was run with dummy variable change in medication [yes/no] to explore effects on main outcome variables. To explore whether the intervention effect differed for T1 and T2, the models with an interaction term for the time points and the condition were compared to models without this interaction term. Likelihood ratio tests for the model fit comparisons were run in package lme4 (Bates et al. 2014) in the statistical environment R (R Core Team 2013).

## Results

**Fig. 1:**
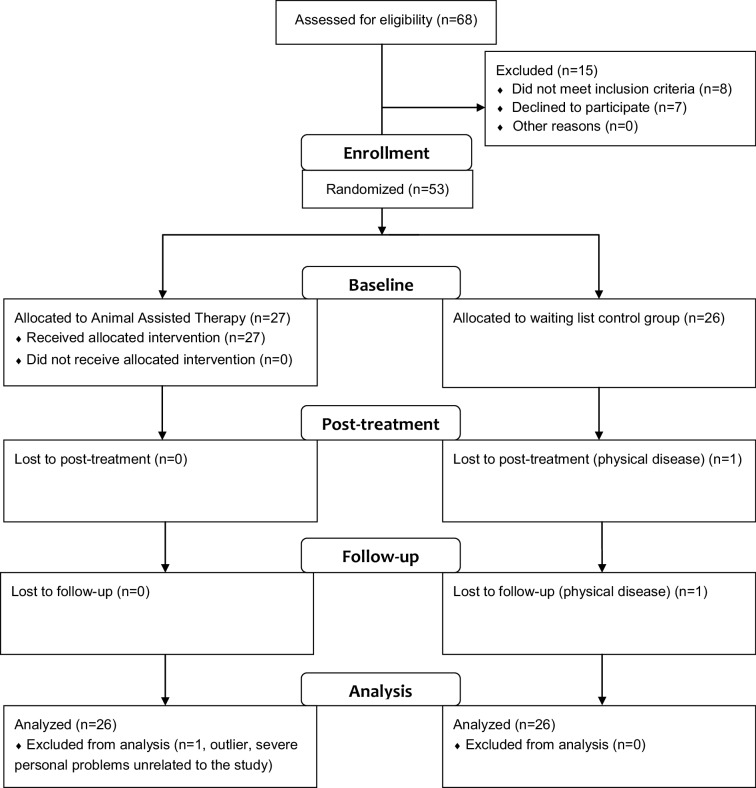
Flowchart design study

In total, 68 respondents from GGZ Oost Brabant with ASD were screened for eligibility; eight were excluded because they did not meet the inclusion criteria, and seven declined to participate (Fig. 1). One participant in the control group dropped out after baseline due to physical illness and the need for intensive revalidation treatment (baseline data were used in analyses). Three measurements of one of the participants were regarded as outliers and excluded from analyses (this was due to multiple personal problems and a stressful life event not related to the study).

Table 1a, b show several baseline characteristics. The average total IQ was 102.1 (SD = 13.7). A total of 18 (35%) participants had a dog at home at T0.

**Table 1:** Baseline characteristics

(a)	
N (%)	
Male	29 (55)
Dog at home at T0	18 (34)
Age, groups (years)	
18–32	19 (36)
33–46	16 (30)
47–60	18 (34)

(b)	Mean	SD	Range	Min	Max
IQ, WAIS III/IV	102.1	13.7	80–160	80	132
Stress, PSS	22.1	6.2	0–40	4	34
Psychological and physical symptoms, SCL-90-R	201.7	56.8	90–450	100	360
Self-esteem, RSES	24.1	5.2	10–40	16	39
Social responsiveness, SRS-A	92.4	25.6	0–192	18	158
Social responsiveness (I), SRS-A (I)	83.9	26.1	0–192	25	163

IQ measured with WAIS-III or WAIS-IV

*PSS* Perceived Stress Scale, *SCL-90-R* symptom checklist, *RSES* Rosenberg Self-Esteem Scale, *SRS-A* social responsiveness

As preplanned, we tested whether the intervention effect might be different for T1 and T2 but, for all tested models, we did not find improvements of the model fit when the interaction term (time-points × intervention condition) was added. Therefore, effects were estimated without this interaction term.

**Fig. 2:**
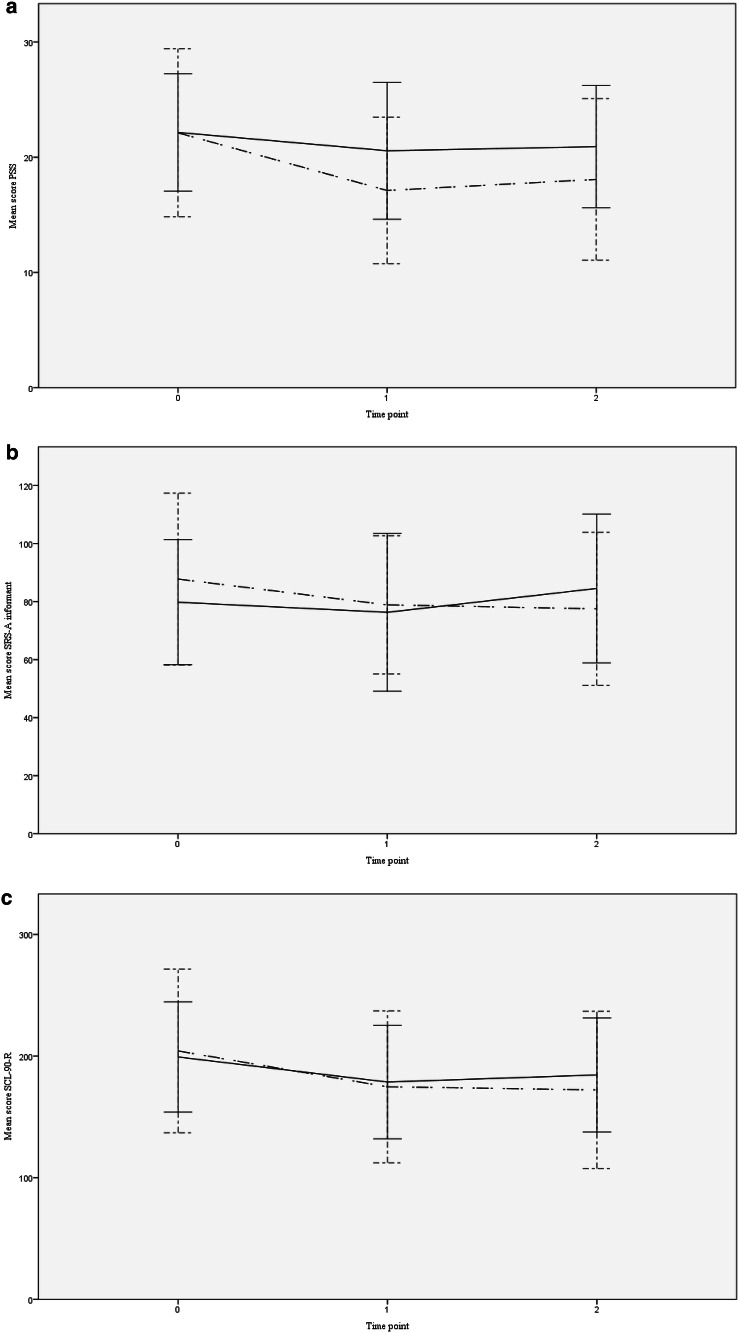

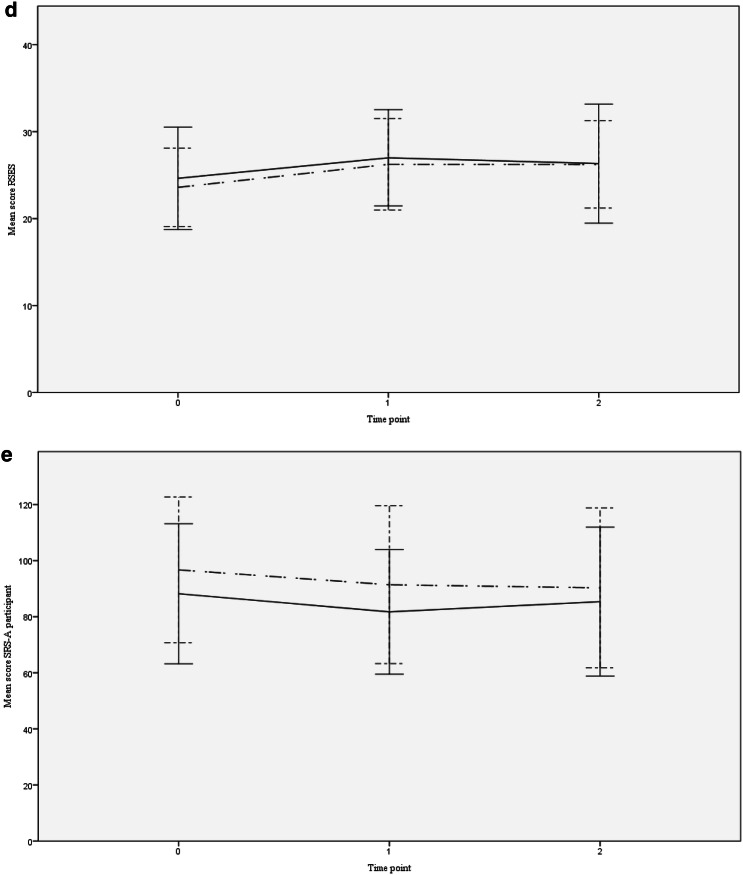
**a** PSS scores. *Notes* mean scores and standard deviations of perceived stress per time point for the intervention and control condition. *PSS* Perceived Stress Scale, Time point 0 = baseline, 1 = post-intervention, 2 = follow-up (dot and dashed line) control condition, (continuous line) intervention condition. **b** SRS-A informant scores. *Notes* mean scores and standard deviations of the impairments in social responsiveness rated by an informant per time point for the intervention and control condition. *SRS-A Informant* Social Responsiveness Scale, informant questionnaire, Time point 0 = baseline, 1 = post-intervention, 2 = follow-up (dot and dashed line) control condition, (continuous line) intervention condition. **c** SCL-90-R scores. Notes: Mean scores and standard deviations of psychological and physical symptoms per time point for the intervention and control condition. SCL-90-R=Symptom Checklist, Time point 0=Baseline, 1=Post-intervention, 2=Follow-up (dot and dashed line) control condition, (continuous line) intervention condition. **d** RSES scores. Notes: Mean scores and standard deviations of self-esteem per time point for the intervention and control condition. *RSES* Rosenberg Self-Esteem Scale, Time point 0 = baseline, 1 = post-intervention, 2 = follow-up (dot and dashed line) control condition, (continuous line) intervention condition. e SRS-A participant scores. Notes: Mean scores and standard deviations of impairments in social responsiveness rated by the participant per time point for the intervention and control condition. *SRS-A Participant* Social Responsiveness Scale, participant questionnaire, time point 0 = baseline, 1 = post-intervention, 2 = follow-up (dot and dashed line) control condition, (continuous line) intervention condition

Both the model with and the model without potential covariates provided a consistent picture of the estimated intervention effects (Table 2). The models showed comparable effect sizes for perceived stress (PSS, estimated effect with potential covariates, − 3.3; 95% CI − 6.1 to − 0.5; *p* = 0.020; *d* = 0.53). Although Fig. 2a demonstrates that stress decreased in the intervention condition at T1, and slightly increased at T2 (while only a small decrease of stress was found in the control condition), the differences in effects for T1 and T2 as compared to T0 were regarded as non-significant (the interaction term did not improve the model fits). Furthermore, a significant intervention effect was shown on impairments in social responsiveness rated by the informant (SRS-A(I), − 11.9; 95% CI − 20.3 to − 3.5; *p* = 0.010; *d* = 0.46). Figure 2b shows a decrease in impairments in social responsiveness in both groups at T1 with an increase in impairments at T2 compared to T0 in the control group, and a slight decrease in impairments at T2 in the intervention group. The significance threshold was not reached for the decrease in psychological and physical symptoms scores (SCL-90-R, − 14.7, 95% CI − 30.8 to 1.4; *p* = 0.072; *d* = 0.26). Intervention effects on self-esteem (RSES, 0.8, 95% CI − 1.3 to 2.9; *p* = 0.440; *d* = 0.16) and deficits in social responsiveness rated by the participant (SRS-A, − 1.3, 95% CI − 7.9 to 5.3; *p* = 0.690; *d* = 0.05) were not significant (respectively; Fig. 2d, e).

Mixed models adjusted for covariates showed small significant intervention effects on the agoraphobia subscale of the SCL-90-R, and small to moderate significant intervention effects on the informant-rated subscales ‘social awareness’ and ‘social communication’ of the SRS-A questionnaire (Table 2). Although a small effect on the depression subscale was significant (d = 0.32, p = 0.042) in the model without the potential covariates, it did not reach the significance threshold (d = 0.33; *p* = 0.055) in the model adjusted for the covariates.

**Table 2:** Intervention effects

Outcomes	Adjusted for potential covariates			Not adjusted basic model			Interaction
	Estimated effect (95% CI)	d	*p* value	Estimated effect (95% CI)	d	*p* value	*p* value
Total scale scores							
PSS	− 3.3 (− 6.1 to − 0.5)	− 0.53	0.020	− 3.3 (− 5.6 to − 0.9)	− 0.53	0.006	0.706
SCL-90-R	− 14.7 (− 30.8 to 1.4)	− 0.26	0.072	− 13.6 (− 28.7 to 1.4)	− 0.24	0.070	0.363
RSES	0.8 (− 1.3 to 2.9)	0.16	0.440	0.5 (− 1.4 to 2.3)	0.09	0.608	0.587
SRS-A	− 1.3 (− 7.9 to 5.3)	− 0.05	0.690	− 0.2 (− 6.5 to 6.1)	− 0.01	0.958	0.208
SRS-A (I)	− 11.9 (− 20.3 to − 3.5)	− 0.46	0.010	− 9.6 (− 17.5 to − 1.8)	− 0.37	0.020	0.380
Subscales of SCL-90-R							
Anxiety	− 1.7 (− 4.5 to 1.0)	− 0.20	0.210	− 1.2 (− 3.7 to 1.2)	− 0.15	0.318	0.752
Agoraphobia	− 1.9 (− 3.4 to − 0.36)	− 0.31	0.016	− 1.4 (− 2.8 to 0.0)	− 0.23	0.056	0.635
Depression	− 4.1 (− 8.3 to 0.1)	− 0.33	0.055	− 4.0 (− 7.8 to − 0.2)	− 0.32	0.042	0.807
Somatization	− 1.3 (− 3.8 to 1.2)	− 0.15	0.314	− 1.3 (− 3.6 to 1.0)	− 0.15	0.260	0.816
Cognitive-performance deficits	− 0.4 (− 3.0 to 2.1)	− 0.06	0.740	− 0.8 (− 3.1 to 1.6)	− 0.10	0.520	0.215
Interpersonal sensitivity and mistrust	− 2.6 (− 6.5 to 1.4)	− 0.19	0.196	− 2.3 (− 6.0 to 1.4)	− 0.17	0.219	0.142
Hostility	− 0.7 (− 2.0 to 0.6)	− 0.20	0.269	− 0.9 (− 2.1 to 0.3)	− 0.24	0.134	0.263
Sleep difficulties	− 0.8 (− 2.0 to 0.4)	− 0.25	0.183	− 0.7 (− 1.8 to 0.4)	− 0.22	0.195	0.912
Subscales of SRS-A informant							
Social awareness	− 3.4 (− 6.2 to − 0.6)	− 0.42	0.019	− 3.2 (− 5.7 to − 0.6)	− 0.39	0.017	0.908
Social communication	− 5.5 (− 9.1 to − 1.9)	− 0.52	0.003	− 4.4 (− 7.7 to − 1.0)	− 0.41	0.011	0.298
Social Motivation	− 1.6 (− 3.5 to 0.4)	− 0.28	0.112	− 0.9 (− 2.7 to 0.9)	− 0.15	0.342	0.114
Restricted interests and repetitive behavior	− 1.4 (− 3.3 to 0.5)	− 0.23	0.141	− 1.0 (− 2.7 to 0.7)	− 0.16	0.263	0.600

The adjusted model shows the estimates for a model corrected, as pre-planned, for potential covariates age (years), gender (male versus female), having a dog at home at T0 (yes/no) and WAIS total IQ, while the basic model shows the estimates for a model without potential covariates. Interaction: models with an interaction term for the time points and the condition were compared to models without this interaction term and *p* values for likelihood ratio tests for the model fit comparisons are shown

*PSS* Perceived Stress Scale, *SCL-90-R* symptom checklist, *RSES* Rosenberg Self-Esteem Scale, *SRS-A* Social Responsiveness Scale for adults, *SRS-A (I)* informant version of the Social Responsiveness Scale for adults

Participants in the age-group between 47 and 60 years old had negligibly higher scores on deficits in social responsiveness, compared to the two younger age groups (18–32 and 33–46 years) (SRS-A, 0.8, 95% CI 0.1 to 1.4; *p* = 0.031; *d* = 0.03). Gender, having a dog at home at T0, and total IQ did not show significant associations with the main outcome variables in any of the models. Additional analyses controlling for change in medication did not reveal changes in effects or a significant difference between those with changes (N = 47) and those with changes (N = 6) in medication.

## Discussion

The results of this exploratory study showed that, compared to the waiting list control group, animal assisted therapy (AAT) with a dog reduced perceived stress and agoraphobia symptoms in adults with ASD. Furthermore, the results implied that AAT reduced impairments in social responsiveness as rated by participants’ spouses, close family members, or friends. There was an indication that depressive symptoms reduced due to the therapy. The analyses implied that these effects, small to medium in size, remained at the 10-week follow-up.

Participant adherence to the therapy program was noteworthy. All participants in the intervention group took part in at least nine out of the ten intervention sessions. Only one recruited participant, who was allocated to the waiting list control group, did not complete the study. The adherence rate (98%) was higher than that reported in other ASD studies (Hesselmark et al. 2014; Turner-Brown et al. 2008; 62–92%). Therefore, it can be argued that the intervention is feasible in adults with ASD who are motivated to receive it.

The reduction of self-perceived stress seen in the intervention condition might be due to AAT’s focus on gaining insight into participants’ own stress levels and finding ways to reduce stress. It could also be that AAT creates a relaxing context for participants. For example, each AAT session in our study included a period of free interaction with a dog. The positive effects of physical contact with an animal are supported by several studies in the general population that showed reduction of self-perceived or physiological stress due to human–animal interactions (Beetz et al. 2012). More specific to ASD, two studies in children with ASD showed reductions of physiological stress confirmed by lower (awakening) cortisol levels (Tabares et al. 2012; Viau et al. 2010). Viau et al. (2010) found that cortisol awakening response decreased in children with ASD after service dogs were placed in their homes. However, this effect disappeared after the dog was removed. In our study the intervention effect remained at the 10-week follow-up. It is plausible that the difference in follow-up effects might be explained by nature of the intervention, e.g. placing a service dog into a home compared to including a therapy dog in a clinical setting. More research is needed to explore the underlying mechanisms of the self-perceived stress reduction and a longer follow-up may shed light on the intervention’s long-term effects.

Although the intervention showed a reduction of psychological and physical problems, this effect did not reach the level of statistical significance for a combined scale score, and the effect-size only slightly outperformed the threshold for clinical significance of d = 0.20 (Cohen 1988).

Remarkably, exploration of subscales showed an intervention effect on agoraphobia. To the best of our knowledge, this variable has not been explored previously in AAT research in children with ASD. A possible explanation for the effect on agoraphobia may be that during the last three sessions of the intervention, participants, accompanied by the therapist and the dog, worked on social fears and controlling environmental stimuli by leaving the mental health facility and practice in the outside world. Social and/or environmental fear in the outside world reflects the main concept of agoraphobia. Another possible explanation for the intervention’s effect on agoraphobia relates to the relationship between stress and agoraphobia (Vreeburg et al. 2010): the reduction in perceived stress may have resulted in a decrease of agoraphobia and fear in social environments, while reduced agoraphobia might likewise reduce perceived stress.

Alongside agoraphobia, models both with and without covariates, showed a reduction in depression scores with clinical relevance (d > 0.20) in the intervention condition. When corrected for covariates, this effect did not reach statistical significance, which may point to some power problems. More research is certainly needed since previous research showed the relation between stress and depression and therefore might indicate that reductions in perceived stress might also reduce depression (Vreeburg et al. 2010). Furthermore, the physical activity and social contact experienced during the therapy sessions might also reduce depressive feelings and thoughts and contribute to a participant’s sense of purpose. In future research, more focus on AAT’s effects on depression can be achieved by using depression-specific instruments and recruiting participants with a clinical diagnosis of depression.

The proxy-reported improvements in social responsiveness are in line with findings in studies in children with ASD. However, these latter studies mainly show improvements on a more basic level of social communication (e.g., eye gazing, making sounds, or speaking words), which were rated using observations and video recordings (O’Haire 2013). Gabriels et al. (2015) used the same instrument used in our study, but to measure social responsiveness in a therapeutic horseback-riding program for children with ASD. They found similar results in the proxy reports and hypothesized that improved social communication skills might be attributed to the unique capacity of animals to serve as a social support or act as a social catalyst in a therapeutic setting. This may make participants more willing to communicate with their environment, leading to improvements in social responsiveness perceived by proxies. These might also have been underlying mechanisms in the adults in our study. However, several other mechanisms, such as modeling and natural (embodied) attunement (synchrony in movement, such as walking speed or eye gazing) might have played a role (Verheggen et al. 2017). More research is needed to examine these hypotheses.

Although we did find an intervention effect in proxy reports on social responsiveness, no effect was found for self-reports of the same outcome. Because adults with ASD may have poor self-awareness (Kievit and Geurts 2011), the validity of their self-reports might be unclear. We suggest using other measures for social responsiveness, such as observational instruments, to explore the differences in results between proxy and self-reports.

Regarding potential covariates in our study, older age was associated with more impairment in social responsiveness. This is in line with Lever and Geurts (2018), who found an age-effect for self-reported ASD traits, with the highest scores in middle adulthood (39–54 years). It was suggested that higher demands such as work, social life, and raising children may explain the age effect (Lever and Geurts 2018).

Results showed no significant intervention effects on self-esteem. Literature shows that interventions directly focusing on improving self-esteem are more effective than interventions focusing on other targets (Haney and Durlak 1998), as it was in our intervention. In contrast to high stress and psychological symptoms, low self-esteem was not an inclusion criterion for participants. In future research, it is important to consider how self-esteem should be targeted in ASD, especially when participants show average levels, as they did in our study.

The study protocol was executed very strictly; study participants were very committed and showed up at every measurement.

A limitation of this study was the relatively small sample size. It is possible, that some of the effects would be significant in a larger sample. Since this study has an exploratory character, a sample size of 30 to 40 participants per condition was regarded as sufficient (Hertzog 2008). Our sample size was comparable to that of other intervention studies in adults with ASD (Sizoo and Kuiper 2017; Spek et al. 2013). Another limitation of the study is that generalization towards the global population might be limited due to lack of ethnic diversity in the sample and exclusion of institutionalized persons, individuals with intellectual disability, and individuals with a fear of dogs.

To the best of our knowledge, this is the first randomized controlled trial that demonstrated benefits of an intervention involving therapy dogs in adults with ASD. Because of its clinically relevant effects and remarkable adherence, AAT can be regarded as a promising therapy for stress-related outcomes in this population. More research is needed on the effects of AAT in the ASD population and with larger sample sizes.
